# A survey of tire-breeding mosquitoes (Diptera: Culicidae) in the Dominican Republic: Considerations about a pressing issue

**DOI:** 10.7705/biomedica.5200

**Published:** 2020-06-30

**Authors:** Mikel A. González, María Altagracia Rodríguez-Sosa, Yohan Enmanuel Vásquez-Bautista, Elizabeth del Carmen Rosario, Jesús Confesor Durán-Tiburcio, Pedro María Alarcón-Elbal

**Affiliations:** 1 Departamento de Sanidad Animal, NEIKER-Instituto Vasco de Investigación y Desarrollo Agrario, Bizkaia, España NEIKER-Instituto Vasco de Investigación y Desarrollo Agrario Bizkaia España; 2 Laboratorio de Entomología, Universidad Agroforestal Fernando Arturo de Meriño (UAFAM), Jarabacoa, República Dominicana Universidad Agroforestal Fernando Arturo de Meriño (UAFAM) Jarabacoa República Dominicana

**Keywords:** Culicidae, Aedes, arboviruses, tires, Dominican Republic, Culicidae, Aedes, arbovirus, neumáticos, República Dominicana

## Abstract

**Introduction:**

Discarded vehicle tires represent a serious threat both to the environment and to public health as they have the potential to harbor important mosquito (Diptera: Culicidae) vectors.

**Objective:**

To assess the importance of used vehicle tires as larval habitats for mosquito fauna that colonize these artificial reservoirs in Jarabacoa, Dominican Republic.

**Materials and methods:**

Used tires were sampled with pipettes at specialized tire fitting shops and scattered stockpiles of tires between June and August, 2018.

**Results:**

We sampled 396 tires; 57 (Container Index=14.4%) were positive for immature stages and contained 2,400 specimens, 11 species, and four genera (*Anopheles*, *Aedes*, *Culex*, and *Toxorhynchites*). The most abundant species was *Aedes albopictus* (42.3%) followed by *Aedes aegypti* (34.3%), and *Culex quinquefasciatus* (14.0%) while other species (9.4%) were less abundant. The container index varied significantly among the different tire sizes (x^2^=13.4; p≤0.05). The highest infestation levels were found in the largest tires. A low positive correlation (r=0.38, n=396; p≤0.001) between the tire size and the prevalence of immature stages was recorded. The presence of organic matter had an overall positive effect on the infestation levels (U=11,430.0; p≤0.001).

**Conclusions:**

These rubber residues, usually located nearby human populations, represent suitable breeding sites for arboviruses vectors such as dengue, chikungunya, Zika, and West Nile.

## Resumen

**Introducción.:**

Los neumáticos usados representan una grave amenaza para el medio ambiente y la salud pública, ya que tienen el potencial de albergar mosquitos (Diptera: Culicidae) vectores de enfermedades.

**Objetivo.:**

Evaluar la importancia de los neumáticos usados como hábitats larvarios de mosquitos que colonizan estos recipientes artificiales en Jarabacoa, República Dominicana.

**Materiales y métodos.:**

Se muestrearon mediante pipetas neumáticos usados en tiendas de venta y reparación y en vertederos entre junio y agosto de 2018.

**Resultados.:**

Se muestrearon 396 neumáticos; 57 (índice de contenedor=14,4 %) albergaban estados inmaduros que contenían 2.400 especímenes de 11 especies y cuatro géneros (*Anopheles*, *Aedes*, *Culex* y *Toxorhynchites*). La especie más abundante fue *Aedes albopictus* (42,3 %) seguida de *Aedes aegypti* (34,3 %) y *Culex quinquefasciatus* (14,0 %), entre otras (9,4 %) de menor abundancia. El índice de contenedor varió significativamente según el tamaño del neumático (x^2^=13,4; p≤0,05) y los mayores niveles de infestación se registraron en los neumáticos de mayor tamaño. Se observó una baja correlación positiva (r=0,38; n=396; p≤0,001) entre el tamaño de neumático y la prevalencia de estadios inmaduros. La presencia de materia orgánica tuvo un efecto positivo sobre los niveles de infestación de mosquitos (U=11430,0; p≤0,001).

**Conclusiones.:**

Estos residuos de caucho, generalmente almacenados cerca de las poblaciones humanas, son propicios para la reproducción de vectores de arbovirus como el dengue, el chikungunya, el Zika y el West Nile.

Management of waste is a demanding and challenging process in developed countries with important implications for human health and well-being, environmental preservation, sustainability, and economy ^1^. Among solid waste, waste or scrap tires (defined as used, discarded, or rejected tires that are either whole or in pieces) represent a serious environmental concern on several fronts: Toxins are released from tire decomposition; incineration or accidental fires can pollute the water, air, and soil ^2^, and abandoned tires or tires stored outside can harbor disease vectors, particularly rodents and mosquitoes (Diptera: Culicidae).

Regarding public health, the trade of used tires has been demonstrated as a mechanism for worldwide dispersal of container-breeding mosquitoes ^3^. This long-distance spread occurs because almost all aedine species lay desiccation-resistant eggs that can persist and survive for long periods away from free water ^4^. When the water level inside tires rises due to rain, the eggs can hatch and facilitate mosquito invasion into new areas ^5^. Discarded tires provide ideal breeding sites as the tire design holds water and offers shade (reduced light) while the rubber creates a suitable environment (safe place) for hatching eggs ^6^.

Service stations, tire dealers, tire repair storage shops, and salvage yards are common sources of discarded tires ^7^. Many tire dealers and tire fitting shops lack appropriate storage sites and, hence, accumulate large quantities of used tires in their facilities and surrounding outdoor areas, which are frequently located near human habitation ^8^. Many mosquito species inhabiting tree holes find these man-made containers a supplement to their natural oviposition sites ^3^ and, therefore, they have the potential to affect the epidemiology of mosquito-borne diseases and become a public health hazard ^9^^,^^10^.

Mosquito-borne diseases represent a serious public health problem in the Americas, especially in the Caribbean, and they are becoming an obstacle to economic development. In the Dominican Republic, several recent studies have identified mosquito-breeding hotspots. Borge de Prada, *et al*. ^11^, and Rodríguez-Sosa, *et al*. ^12^, showed that the accumulation of waste in public spaces allows the proliferation of mosquitoes of public health importance and Rodríguez-Sosa, *et al*. ^13^, also observed this problem in the domestic environment. On the other hand, González, *et al*. ^14^, showed the relevance of artificial containers as important urban foci with a high density of vector mosquito species in cemeteries.

However, such studies are still limited in the Caribbean region. In this context, the objective of our study was to assess the importance of used vehicle tires as larval habitats for mosquito fauna by evaluating species composition and relative abundance and correlating mosquito numbers with attributes such as tire-size class, container index, and presence or absence of decaying detritus in periurban areas of the Dominican Republic.

## Materials and methods

### Study area

We conducted the study in the municipality of Jarabacoa (La Vega Province, Dominican Republic) whose population is about 32,600 inhabitants within an area of 660 km^2^, approximately. Jarabacoa has a typical tropical rainforest climate with 1,340 mm of annual rainfall and an annual daily mean temperature of 22.9 °C; its Koppen climate classification is Af. The city is situated in a strategic position for the development of mountain tourism and ecotourism.

### Data collection

**Figure 1:**
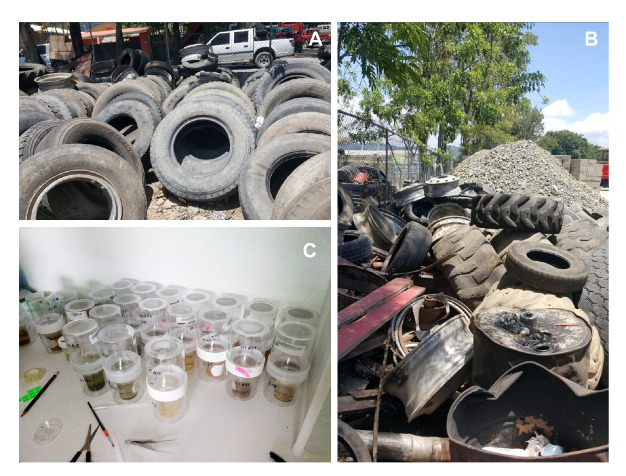
Sources of discarded vehicle tires and mosquito breeders in the laboratory. A) Used car tires stacked in a service station against a fence. B) Mixed vehicle tires abandoned in a fitting shop. C) Mosquito breeders for rearing pupae to the adult stage.

We tried to locate the majority of sources and accumulation sites of used vehicle tires. Discarded tires were surveyed from June to August, 2018, at four main tire repair storage shops (known as *gomeras* by locals) with piles of tires accumulated outdoors, as well as two sites containing two and three discarded tires, respectively, located in public spaces (figure 1A-B).

Informed consent was obtained from each of the sampled private shop owners.

All water-filled tires (positive or negative) were counted and classified according to the tire size in four classes: motorcycle, car, truck, and tractor tires with a mean tire rolling diameter of 64, 76, 102, and 178 cm, respectively. The presence of organic matter was recorded as positive (input of decaying leaves, plants, or fine detritus) or negative (no organic matter or just a thin layer of it) in water-filled tires. If present, all mosquito larvae and/or pupae were collected from each tire into plastic trays and then transferred to hermetic tubes using disposable pipettes. No tire was sampled more than once.

In the laboratory, immature stages of mosquitoes were transferred alive into mosquito breeders (Bioquip Products, USA) with their own water (figure 1C). Collected larvae were killed by placing them in hot water (60 °C) for 1 min once they reached the IV instar, after which they were fixed in 70% ethanol. Pupae were raised to adults and then killed by freezing to enable accurate identification. Both larvae and adults were identified using the key published by González-Broche ^15^. Voucher specimens were deposited in the Laboratory of Entomology at *Universidad Agroforestal Fernando Arturo de Meriño* (Jarabacoa, Dominican Republic).

### Statistical analysis

We evaluated the influence of tire-size class on the container index and the prevalence of immature stages. We ran the analysis using non-parametric tests after determining that the data were not normally distributed. The container index was calculated as the number of positive tires/number of sampled tires * 100.

We compared the container index for the four classes of tires using the chi- squared (x^2^) test followed by pairwise comparisons. The Spearman correlation coefficient was calculated to assess the association between tire-size classes and mosquito prevalence. Data are presented as medians (range). The effect of the presence of organic matter inside water-filled tires on the immature mosquitoes was assessed by Mann-Whitney U tests. For the descriptive analysis and statistics, we used the IBM SPSS^™^, version 22.0, software (alpha-level=0.05).

## Results

Out of the 396 used vehicle tires sampled, 227 (57.4%) were water- filled tires and 57 (container index=14.4 %) harbored immature stages of mosquitoes. In total, 2,400 immature specimens (2,279 larvae and 121 pupae) of 11 mosquito species and four genera (*Anopheles, Aedes, Culex,* and *Toxorhynchites*) were collected and subsequently identified (table 1). *Aedes* (*Stegomyia*) *albopictus* (Skuse) accounted for most specimens (1,017 specimens, 42.3%) followed by *Aedes* (*Stegomyia*) *aegypti* (Linnaeus) (825 specimens, 34.3%) and to a lesser extent, *Culex* (*Culex*) *quinquefasciatus* Say (336 specimens, 14.0%). The remaining 9.4% belonged to eight other mosquito species that were heterogeneously distributed (table 1).

**Table 1:** Summary of information related to the immature mosquitoes collected from vehicle tires in Jarabacoa, Dominican Republic (June-August 2018)

Attributes	Tire-size class				
	Motorcycle	Car	Truck	Tractor	Total
Tire rolling diameter (Ø, cm)	64	76	102	178	-
Sampled tires (n)	11	319	56	10	396
Positive tires (n)	0	39	14	4	57
Container index (%)	0	12	25	40	14
Water-filled tires (n)	9	158	54	6	227
Organic matter in water-filled tires (%)	0	29	9	4	42
Specimens (n)	0	1,179	790	431	2,400
Median specimens/tire (range)	0 (0)	16.0 (0-216)	39.5 (0-163)	119.0 (0-145)	24.0 (0-216)
Species richness	0	11	2	4	11

Tires containing immature mosquitoes had a median of 24 (216) specimens/ tire. The percentage of water-filled tires varied from 49% (in car tires) to 81% (in motorcycle tires) (table 1). Most of the used vehicle tires were from cars (80.1%) followed by trucks (14.1%) and the remaining classes (≤6%). The lowest species richness was found in motorcycle tires with no Culicidae specimens while the highest was found in car tires containing all species collected (table 1).

The container index varied significantly among the different tire-size classes (x^2^= 3.4; df=3; p=0.04) with significantly higher prevalence rates for truck (x^2^=18.2; p=0.032) and tractor tires (x^2^=11.8; p=0.028) than for car tires. The highest container index was recorded in truck tires (container index=40%) and the lowest in motorcycle tires (container index=0%) (table 2). Although recorded in lower numbers, *A. aegypti* accounted for a higher total container index than its congener *A. albopictus* (table 2).

**Table 2:** Mosquito species identified in different types of discarded tires in Jarabacoa, Dominican Republic (June-August 2018)

Species	Tire-size class *					
	Tractor	Car	Truck	Tractor	Total	
	n (%)	n (%)	n (%)	n (%)	n (%)	CI (%)^*^
*C. quinquefasciatus*	0	240 (20.3)	62 (7.8)	34 (7.9)	336 (14.0)	4.3
*C. corniger*	0	7 (0.6)	0	0	7 (0.3)	0.2
*C. atratus*	0	14 (1.2)	0	0	14 (0.6)	0.2
*C. secutor*	0	6 (0.5)	0	0	6 (0.2)	0.5
*C. nigripalpus*	0	2 (0.2)	0	71 (16.5)	73 (3.0)	0.7
*T. portoricensis*	0	70 5.9)	0	0	70 (3.0)	0.2
*A. albonotatus*	0	47 (4.0)	0	0	47 (1.9)	1.3
*A. aegypti*	0	313 (26.5)	300 (38.0)	212 (49.2)	825 (34.3)	11.6
*A. albopictus*	0	467 (39.6)	436 (55.2)	114 (26.4)	1,017 (42.3)	7.6
*An. grabhamii*	0	1 (0.1)	0	0	1 (0.1)	0.2
*An. crucians*	0	4 (0.3)	0	0	4 (0.2)	0.2
Total	0	1,179	790	431	2,400 (100)	14.4

* Container Index

We registered a low positive correlation (r=0.38, p≤0.001) between the tire-size class and the prevalence of immature stages. Overall, the immature mosquito prevalence rates varied significantly according to the vehicle tire-size class (x^2^=18.2; p≤0.001). A higher number of specimens was found in tractor tires. Data is presented as medians with its raw range value [median: 119 (range: 0, 145) compared to car tires (median=16 (range: 0, 216), truck tires (median=39.5 (range: 0, 163), and motorcycle tires (median=0 (range: 0, 0)] (table 1).

Forty-two (74% of the total sampled tires) of the inhabited tires contained organic matter. The presence of organic matter (in 26 of 216 tires while no organic matter was found in 16 of 105) inside discarded tires had a positive effect on the prevalence of immature stages of mosquitoes (U=11,430.0, p≤0.001). This trend was observed for the three other most common species collected (*Aedes aegypti, A. albopictus,* and *C. quinquefasciatus*).

## Discussion

To our knowledge, this survey represents the most comprehensive study published in the region about used vehicle tires. Our results draw attention to some important aspects of vector distribution. There has recently been increasing interest in the study of vectors whose breeding sites are located in environments with accumulated rubbish and waste ^16^. In Central America, studies on mosquito fauna in discarded tires are relatively common ^17^^-^^20^ and all of them showed the relevance of tire-breeding habitats as an important locus for mosquito species of public health significance. In the Dominican Republic, used tires have also been reported as one of the key containers for mosquito breeding to a greater or lesser degree ^11^^-^^13^^,^^21^^-^^24^.

Our survey revealed important points:

- Tires support a high species richness comprising nearly 70% of the known species spotted in the studied municipality;
- Moderate infestation levels of *A. aegypti* and *A. albopictus* immature stages were recorded in these particular breeding habitats consistent with literature published elsewhere ^25^^,^^26^;
- Mosquito container indexes varied significantly across the four tiresize classes: lower levels of infestation were found in smaller tires (motorcycles) compared to larger tires (truck and tractor) as reported by McMahon, *et al*. ^9^, who, in turn, noted that bigger tires had more surface and stored water volumes of up to 200 L ^7^, therefore being more prone to serve as breeding sites for mosquitoes;
- The overall container index obtained in our study was similar to that reported in other regions such as Argentina (total mosquitoes, container index=17.7; container index=11.6 for *A. aegypti*) ^27^, and
- The presence of organic matter in discarded tires had a positive effect on the number of immature mosquitoes in line with other studies ^28^^,^^29^ thus corroborating the contribution of organic matter (detritus, leaves, twigs, seeds, etc.) to larval nutrition resulting in larger females and more specimens.

*Aedes aegypti* and *A. albopictus* are two aedine mosquito species of cosmopolitan distribution, which prefer laying their eggs in artificial containers like discarded tires. In fact, they are called «tire-breeding mosquitoes» since in many cases these containers are among their preferred breeding habitats ^30^^,^^31^. On the Hispaniola island, water storage tanks were the most frequent container used by *A. aegypti* followed by used car tires ^32^. In the Dominican Republic, discarded tires are important for the proliferation of *Aedes* spp. and *Culex* spp. both in domestic and public environments ^11^^,^^13^. In this regard, *Culex*, mainly *C. quinquefasciatus*, is frequently reported as a common tire-breeding mosquito in different countries of the Caribbean and Latin America ^27^^,^^33^.

In addition to *Aedes*, other species of anopheline mosquitoes may be dispersed through human activities, e.g., movement of used vehicle tires ^34^. We found *Anopheles (Anopheles) grabhamii* Theobald and *Anopheles* (*Anopheles*) *crucians* Weidemann breeding in one single car tire. *Anopheles* (*Nyssorrhynchus*) *albimanus* Weidemann, known as the main vector of malaria in Central America including the Caribbean territory, had been previously reported breeding in used tires in the municipality ^11^ but was not found in the present study.

These findings reveal that anopheline species, generally found in different types of natural ecosystems ^14^^,^^35^, may also breed in artificial containers albeit with low frequency, which might explain why they are found in urban areas as reported by Mendizäbal-Alcalä, *et al.*^36^. On the other hand, the presence of *Toxorhynchites* (*Lynchiella*) *portoricensis* (von Röder) was occasional, although it seems to breed in tires more frequently than Dominican *Anopheles* spp. as noted in the literature ^12^^,^^15^.

Waste management regulations are country-specific and should be implemented to promote the environmentally sound management of waste tires by providing a regulatory framework. According to Resolution No. 005-2015 of the Ministry of Environment and Natural Resources of the Dominican Republic, discarding used tires in unauthorized dumps, streams, and watercourses, as well as in other places such as wasteland and backyards, is prohibited to avoid the accumulation of water and propagation of vectors ^37^. Despite this, discarded tires improperly stockpiled or illegally dumped in the country are common. This situation is worrying, especially at service stations, tire dealers, auto repair shops, tire fitting shops, and salvage yards, many of which lack a registered waste tire storage site. These businesses have large accumulations of waste tires stored outside their facilities, sometimes even on the roof and in surrounding areas, as can be observed along the Duarte Highway on the way to Santo Domingo (the capital city of the Dominican Republic).

Considering the almost 7 billion people in the world with 1.1 billion vehicles on the road, 1.7 billion new tires produced per year, and up to 1 billion waste tires generated per year, the recycling and reuse of rubber should be a human obligation. The recycling of waste tires has been widely studied over recent decades, with applications in infrastructure and civil construction, especially the production of asphalt, concrete, and isolators for lightweight construction, waterproofing systems, and membrane liners, among others ^38^^,^^39^. Nowadays, inexpensive and eco-friendly hand-made alternatives are becoming more common, for example, the conversion of scrap tires into furniture, decorations, toys, flower pots, etc., as an effective way for community renewal initiatives to promote artisanal handicrafts.

In conclusion, among artificial containers, discarded vehicle tires represent an important source of mosquito vectors in many countries including the Dominican Republic. Despite international and national guidelines warning about the proper disposal and storage of vehicle tires, it seems clear that this urban solid waste is not being considered a serious threat in mosquito control activities in the Dominican Republic as it does in other places ^27^.

Therefore, it is imperative that regulations are implemented to manage this waste both at the municipal and national levels in line with other countries ^40^. We also recommend that preventive measures be strengthened via awareness campaigns aimed at informing, sensitizing, and mobilizing communities to eliminate the risks associated with the presence of mosquito- borne diseases as was recently implemented in the municipality of Jarabacoa by Vásquez-Bautista, *et al*. ^41^. It will also be essential to undertake a more thorough cleaning of wastelands to reduce health risks related to the presence of mosquitoes. Ultimately, the practice of waste reduction, reuse, and recycling is strongly recommended.

## References

[1] World Health Organization (2019). Waste and human health: Evidence and needs. WHO Meet Report.

[2] Environment Protection Authority (2019). Whaste Guidelines.

[3] Reiter P, Sprenger D (1987). The used tire trade: A mechanism for the worldwide dispersal of container breeding mosquitoes. J Am Mosq Control Assoc.

[4] Clements A (1992). The Biology of Mosquitoes. Volume 1. Development, Nutrition, and Reproduction.

[5] Beeuwkes J, den Hartog W, Dik M, Scholte EJ (2011). Surveillance and findings of exotic mosquitoes in used tires in The Netherlands: A methodological approach.

[6] Eshag OS, Bashir NH, Dukeen MY (2019). Prevalence, habitat and productivity profiles of Aedes mosquitoes (Diptera: Culicidae) in Sennar state, Sudan. Int J Res Mosq.

[7] Baumgartner DL (1988). Suburban accumulations of discarded tires in northeastern Illinois and their associated mosquitoes.

[8] Yee DA, Kneitel JM, Juliano SA (2010). Environmental correlates of abundances of mosquito species and stages in discarded vehicle tires. J Med Entomol.

[9] McMahon TJ, Galloway TD, Anderson RA (2008). Tires as larval habitats for mosquitoes (Diptera: Culicidae) in southern Manitoba.

[10] Dinh ETN, Novak RJ (2018). Diversity and abundance of mosquitoes inhabiting waste tires in a subtropical swamp in urban Florida.

[11] Borge-de Prada M, Rodríguez-Sosa MA, Vásquez-Bautista YE, Guerrero KA, Alarcón-Elbal PM (2018). Mosquitos (Diptera, Culicidae) de importancia médica asociados a residuos sólidos urbanos en Jarabacoa, República Dominicana. Rev Salud Jalisco.

[12] Rodríguez-Sosa MA, Rueda J, Vásquez-Bautista YE, Fimia-Duarte R, Borge-de Prada M, Guerrero KA (2019). Diversidad de mosquitos (Diptera: Culicidae) de Jarabacoa, República Dominicana.

[13] Rodríguez-Sosa MA, Diéguez-Fernández L, Borge-de Prada M, Vásquez-Bautista YE, Alarcón-Elbal PM (2019). Sitios de cría de Aedes albopictus (Skuse) (Diptera: Culicidae) en el entorno doméstico en Jarabacoa, República Dominicana. Rev Chil Entomol.

[14] González MA, Rodríguez-Sosa MA, Vásquez-Bautista YE, Diéguez-Fernández L, Borge-de Prada M, Guerrero KA (2019). Micro-environmental features associated to container-dwelling mosquitoes (Diptera: Culicidae) in an urban cemetery of the Dominican Republic. Rev Biol Trop.

[15] González-Broche R (2006). Culícidos de Cuba.

[16] Onyido AE, Okolo PO, Obiukwu MO, Amadi ES (2009). A survey of vectors of public health diseases in un-disposed refuse dumps in Awka Town, Anambra state, southeastern Nigeria. Res J Parasitol.

[17] Zapata-Peniche A, Manrique-Saide P, Rebollar-Téllez EA, Che-Mendoza A, Dzul-Manzanilla F (2017). Identificación de larvas de mosquitos (Diptera: Culicidae) de Mérida, Yucatán, México, y sus principales criaderos. Rev Biomed.

[18] Alcalá L, Quintero J, González-Uribe C, Brochero H (2015). Productividad de *Aedes aegypti* (L .) (Diptera: Culicidae) en viviendas y espacios públicos en una ciudad endémica para dengue en Colombia.

[19] Maestre-Serrano R, Pacheco-Lugo L, Salcedo-Mendoza S (2015). Índices de infestación aédica e identificación de conocimientos, actitudes y prácticas sobre dengue en llanterías del departamento del Atlántico, Colombia. Rev Salud Pública.

[20] Monzón MV, Rodríguez J, Diéguez L, Alarcón-Elbal PM, San Martín JL (2019). Hábitats de cría de Aedes aegypti (Diptera: Culicidae) en Jutiapa, Guatemala. Novitates Caribaea.

[21] Peña CJ, Zaglul A (1986). Los mosquitos de la ciudad de Santo Domingo. Cienc. Soc.

[22] Peña CJ, Gonzálvez G, Chadee DD (2003). Seasonal prevalence and container preferences of Aedes albopictus in Santo Domingo City, Dominican Republic. J Vector Ecol.

[23] Peña CJ, Gonzálvez G, Chadee DD (2004). A modified tire ovitrap for monitoring Aedes albopictus in the field. J Vector Ecol.

[24] Tidwell MA, Williams DC, Carvalho Tidwell T, Peña CJ, Gwinn TA, Focks DA (1990). Baseline data on Aedes aegypti populations in Santo Domingo, Dominican Republic. J Am Mosq Control Assoc.

[25] Roiz D, Eritja R, Escosa R, Lucientes J, Marquès E, Melero-Alcíbar R (2007). A survey of mosquitoes breeding in used tires in Spain for the detection of imported potential vector species. J Vector Ecol.

[26] Stein M, Oria GI, Almirón WR (2002). Principales criaderos para Aedes aegypti y culícidos asociados, Argentina. Rev Saúde Pública.

[27] Rubio A, Cardo MV, Vezzani D (2011). Tire-breeding mosquitoes of public health importance along an urbanisation gradient in Buenos Aires, Argentina. Mem Inst Oswaldo Cruz.

[28] Yee DA (2008). Tires as habitats for mosquitoes: A review of studies within the eastern United States. J Med Entomol.

[29] Kling LJ, Juliano SA, Yee DA (2007). Larval mosquito communities in discarded vehicle tires in a forested and unforested site: Detritus type, amount, and water nutrient differences. J Vector Ecol.

[30] Ferede G, Tiruneh M, Abate E, Kassa WJ, Wondimeneh Y, Damtie D (2018). Distribution and larval breeding habitats of Aedes mosquito species in residential areas of northwest Ethiopia. Epidemiol Health.

[31] Getachew D, Tekie H, Gebre-Michael T, Balkew M, Mesfin A (2015). Breeding sites of Aedes aegypti : Potential dengue vectors in Dire Dawa, East Ethiopia. Interdiscip Perspect Infect Dis.

[32] Marquetti MC, Fuster C, Ponce F, Estévez G, Somarriba L (2011). Estudio descriptivo de la distribución y de la positividad larvaria de Aedes aegypti (Diptera : Culicidae) en Haití. Rev Biomed.

[33] Bisset-Lazcano JA, Marquetti-Fernández MC, Rodríguez-Coto MM (2017). Contribución de estudios entomológicos sobre Aedes aegypti y Aedes albopictus: retrospectiva y retos para su control en Cuba. Rev Cubana Med Trop.

[34] Head GP, Savinelli C (2014). Adapting insect resistance management programs to local needs. In: Insect resistance management. Biology, Economics, and Prediction.

[35] Valdés-Miró V, Marquetti-Fernández MC (2010). Presencia larval de Anopheles albimanus (Diptera: Culicidae) en el municipio Boyeros. Rev Cubana Med Trop.

[36] Mendizábal-Alcalá ME, Peraza-Cuesta I, Pérez-Castillo M, Valdés-Miró V, Molina-Torriente RE, Marquetti-Fernández MC (2014). Presencia larval de Anopheles albimanus (Diptera: Culicidae) en La Habana, Cuba 2010-2012. Rev Cubana Med Trop.

[37] Ministerio de Medio Ambiente y Recursos Naturales (2015). Resolución No. 005-2015. Que aprueba y emite el reglamento técnico ambiental para la gestión de neumáticos fuera de uso.

[38] Yilmaz A, Degirmenci N (2009). Possibility of using waste tire rubber and fly ash with Portland cement as construction materials. Waste Manag.

[39] Peláez-Arroyave GJ, Velásquez-Restrepo SM, Giraldo-Vásquez DH (2017). Aplicaciones de caucho reciclado: una revisión de la literatura. Cienc Ing Neogranadina.

[40] Novak RJ (1995). A North American model to contain the spread of Aedes albopictus through tire legislation. Parassitologia.

[41] Vásquez-Bautista YE, Hernández-Barrios Y, Rodríguez-Sosa MA, del Carmen Rosario E, Durán-Tiburcio JC, Alarcón-Elbal PM (2019). «Sácale los pies al mosquito»: resultados parciales de la implementación de un programa educativo en República Dominicana. Cienc Soc.

